# Diseases of the Blood

**Published:** 1905-07-15

**Authors:** 


### Progress in Medicine and Surgery.

### DISEASES OF THE BLOOD.

(*Continued from page 263.*)

**Bone Marrow.**—An excellent method of microscopical examination of the bone marrow is described by Price Jones,[3] who remarks that the results obtained from smear preparations and sections are unsatisfactory. The marrow is obtained, as soon as possible after death, from a rib or vertebra by squeezing it out of the bone by means of a strong forceps or scissors. It is conveniently collected on a platinum loop and transferred at once to a watch-glass containing the dissociating reagent. There results a more or less complete emulsion, and the cellular elements of the marrow are dissociated. The dissociating reagent is prepared by diluting glycerine with ammonia-free distilled water to form a 10 per cent. solution and titrating this against decinormal sodium hydrate, using phenolphthaleim as an indicator. A loopful of 10 per cent. glycerine is then placed on a clean cover slip, and to this a loopful of the marrow emulsion is added and then spread over the surface of the slip. The film thus prepared is allowed to dry in the air; it is then fixed and stained by Jenner's stain—treated, in fact, like a blood film. On microscopical examination the various cells should be seen well separated from one another. The cells seen are the myelocytes or marrow cells proper, and those cells which belong to the blood. The red cells comprise the non-nucleated and the nucleated red cells. In normal marrow the proportions of these are about 10 to 1. The white cells are the non-granular—comprising giant or mother cells and lymphoid cells—and the granular, the coarse oxyphile cells, fine oxyphile cells, amphiphile cells, coarse and fine basophile cells, and polymorphonuclear cells. In healthy man the percentage counts are giant cells ·2, lymphoid cells 50·2, coarse oxyphile cells 3·3, fine oxyphile cells 39·3, no amphiphile or coarse basophile cells, polymorphonuclear cells 6·7.

**Diseases of the Blood-forming Organs.**—In a review of recent investigations on diseases of the blood-forming organs, Shaw[4] says that the marrow contains "lymphoid" cells which give rise to various members of the leucoblastic series, and in health to more granular than non-granular cells. In all cases of leucocythemia the marrow is affected, whereas the spleen and lymphatic tissues may or may not be affected. The myeloid tissue may produce cells which are mostly myelocytic in type, or mostly lymphocytic in type. Hence in any type of leucocythemia we may meet with many degrees of preponderance of one or other cell, or actually find a change in the type of cell present. It is possible that the pathogenesis of lymphadenoma, or Hodgkin's disease, may be of the same nature as that of leucocythemia, differing in degree only. As a rule, of course, the blood picture in cases of lymphadenoma shows no change, or one merely of a proportional increase in lymphocytes, but such conditions have been known to pass into cases of lymph- or myelo-cytic leucocythemia. In regard to this question it is interesting to note that whilst some observers have found changes in the marrow in all cases of lymphadenoma, other observers say that the marrow changes are not always found. Various exceptional cases of acute leucocythemia have been recorded lately—*e.g.* one in which there was a transitory paresis of the face muscles; two, by Taylor, in which there was the unusual feature of great enlargement of various secretory glands—parotid, submaxillary, liver, kidney mammæ—and one in which there were numerous subcutaneous lymphocytic nodules. Melland has published the account of a case of leucocythemia with change of type from the splenomedullary to the lymphatic form. When the patient first came under observation there was a very large spleen, and no enlargement of the lymphatic glands, whilst the blood contained 22 per cent. of myelocytes and 46 per cent. of large lymphocytes. Later on these proportions changed, and a differential count gave 4 per cent. of myelocytes and 83 per cent. of large lymphocytes. Post-mortem, the lymphatic glands showed no enlargement or evidence of hyperactivity, the spleen was large, and the bone marrow consisted almost entirely of cells similar to the large lymphocytes in the blood.

**Acute Myelocytic Leukæmia** is rare, as opposed to the comparative frequency of acute lymphatic leukæmia, and a case—the first in Great Britain—is published by Elder and Fowler.[6] The total duration of the case was less than six weeks. A previously healthy man, aged 34, developed general asthenia comparatively suddenly and without apparent cause. The most prominent early symptom was the enfeebled and rapid cardiac action. Anæmia was at first masked by the naturally ruddy complexion. A trifling erosion of the gums, hæmorrhages from the stomach, bowel, and nose, associated with a slight degree of fever and progressive weakness, completed the picture. The total leucocytes gradually increased from 7,210 to 20,000 per cubic mm. On differential counting, lymphocytes were about 28 per cent., polynuclears about 55 per cent., eosinophiles 3 per cent., and myelocytes 13 per cent. Nucleated reds were also abundant. The diagnosis was unquestionable; the presence of so large a proportion of myelocytes as 13 per cent., even in the absence of any marked leucocytosis, pointed conclusively to the case being one of myelocytic leukæmia, whilst the short course, with hæmorrhagic symptoms, conforms exactly to the type of acute leukæmia. The diagnosis was confirmed by the pathological changes found after death—proliferation of the myelocytes of the bone marrow and myelocytic infiltration of the spleen. In medical literature only six undoubted cases of this condition are to be found. On comparison of the records of these, and of this seventh case, it may be concluded that the course and clinical features of acute myelocytic leukæmia are practically identical with those of the much more common acute lymphatic leukæmia. The anæmia is generally greater than in chronic cases. The leucocyte count is not always high, and may be little above normal. Eosinophiles may be absent or few, and the same is to an even greater extent true of mast cells.

[3] Price Jones, Brit. Med. Jour., Feb. 25. [4] Shaw, Practitioner, October. [5] Melland, Lancet, Feb. 25. [6] Elder and Fowler, Edin. Med. Jour., December.

*(To be concluded.)*

